# The Effectiveness of Pilates Exercise in People with Chronic Low Back Pain: A Systematic Review

**DOI:** 10.1371/journal.pone.0100402

**Published:** 2014-07-01

**Authors:** Cherie Wells, Gregory S. Kolt, Paul Marshall, Bridget Hill, Andrea Bialocerkowski

**Affiliations:** 1 Faculty of Health, University of Canberra, Bruce, Australian Capital Territory, Australia; 2 School of Science and Health, University of Western Sydney, Campbelltown, New South Wales, Australia; 3 Epworth HealthCare, Richmond, Victoria, Australia; 4 Griffith Health Institute, Griffith University, Gold Coast, Queensland, Australia; The University of Queensland, Australia

## Abstract

**Objective:**

To evaluate the effectiveness of Pilates exercise in people with chronic low back pain (CLBP) through a systematic review of randomised controlled trials (RCTs).

**Data Sources:**

A search for RCTs was undertaken using Medical Search Terms and synonyms for “Pilates” and “low back pain” within the maximal date range of 10 databases. Databases included the Cumulative Index to Nursing and Allied Health Literature; Cochrane Library; Medline; Physiotherapy Evidence Database; ProQuest: Health and Medical Complete, Nursing and Allied Health Source, Dissertation and Theses; Scopus; Sport Discus; Web of Science.

**Study Selection:**

Two independent reviewers were involved in the selection of evidence. To be included, relevant RCTs needed to be published in the English language. From 152 studies, 14 RCTs were included.

**Data Extraction:**

Two independent reviewers appraised the methodological quality of RCTs using the McMaster Critical Review Form for Quantitative Studies. The author(s), year of publication, and details regarding participants, Pilates exercise, comparison treatments, and outcome measures, and findings, were then extracted.

**Data Synthesis:**

The methodological quality of RCTs ranged from “poor” to “excellent”. A meta-analysis of RCTs was not undertaken due to the heterogeneity of RCTs. Pilates exercise provided statistically significant improvements in pain and functional ability compared to usual care and physical activity between 4 and 15 weeks, but not at 24 weeks. There were no consistent statistically significant differences in improvements in pain and functional ability with Pilates exercise, massage therapy, or other forms of exercise at any time period.

**Conclusions:**

Pilates exercise offers greater improvements in pain and functional ability compared to usual care and physical activity in the short term. Pilates exercise offers equivalent improvements to massage therapy and other forms of exercise. Future research should explore optimal Pilates exercise designs, and whether some people with CLBP may benefit from Pilates exercise more than others.

## Introduction

Chronic low back pain (CLBP) is defined as pain for more than twelve weeks in the posterior lumbar region between the twelfth ribs and inferior gluteal folds [1]. CLBP is highly prevalent and associated with significant levels of disability [2]–[4]. As a consequence, CLBP places a large social and economic burden on society [2]–[4].

Pilates exercise is a commonly prescribed to people with CLBP [5]–[7]. Pilates exercise is named after its founder, Joseph Pilates, who developed a series of exercises in the 1920s to encourage physical and mental conditioning [8], [9]. Core stability, strength and flexibility are emphasised in Pilates exercise, as is control of movement, posture, and breathing [9]. All of these aspects of Pilates exercise may benefit people with CLBP as exercises with similar features have been successful in reducing pain and improving functional ability [10]–[12].

When treating people with CLBP, it has been suggested in a Delphi survey that supervised Pilates exercise sessions should be undertaken 2–3 times per week for 3–6 months, and be supplemented by home exercises [13]. Individualised supervision has been advised in the first 2 weeks, but thereafter group sessions of up to 4 clients per therapist [13]. The use of specialised Pilates exercise equipment with spring resistance, such as a Reformer, has also been recommended for people with CLBP [13], [14].

Despite the popularity of Pilates exercise in treating people with CLBP, its effectiveness in people with CLBP is yet to be established [15]. Six systematic reviews have investigated the effectiveness of Pilates exercise in people with CLBP, and a protocol for a Cochrane review has also been published [6], [7], [16]–[20]. Completed reviews, though, report different findings [6], [7], [16]–[20]. Several reviews report a decrease in pain, but not all report improvements in functional ability [6], [16]–[18]. Other reviews report no improvement in pain or functional ability or inconclusive findings [7], [13], [19]. The small number and mixed methodological quality of primary studies has made reporting of credible results difficult [15]. Several reviews have also conducted meta-analyses in the presence of significant clinical heterogeneity, resulting in misleading findings [15].

Recently, several randomised controlled trials (RCTs) have been published that are relevant to evaluating the effectiveness of Pilates exercise in people with CLBP [21]–[26]. The majority of these RCTs have not been included in prior reviews, so incorporating this new evidence in an updated systematic review is indicated. Given there is now a moderate volume of evidence available, it is also appropriate that this new systematic review includes only RCTs. This will ensure this review represents a high level of evidence and increases the credibility of results [15], [27], [28].

The aim of the following systematic review is to provide an update on the effectiveness of Pilates exercise in reducing pain and improving functional ability of people with CLBP based on the highest level and quality of research evidence available [28].

## Materials and Methods

### Study Design

A systematic review was undertaken to locate, evaluate and summarise findings from RCTs that have investigated the effectiveness of Pilates exercise in people with CLBP. A systematic review was chosen over a narrative review as it limits bias and error in the selection and appraisal of evidence [29], [30]. In this systematic review, a comprehensive search of the literature was undertaken to answer a focused question, the methodological quality of primary studies was appraised, and findings were synthesised to address the study aim [29], [30].

### Search Strategy

A comprehensive literature search for evidence was undertaken on the 1st May, 2014 using 10 databases: Cumulative Index to Nursing and Allied Health Literature, Cochrane Library, Medline, Physiotherapy Evidence Database, ProQuest: Health and Medical Complete, Proquest: Nursing and Allied Health Source, Proquest: Dissertation and Theses, Scopus, Sport Discus, and Web of Science. To ensure relevant trials were not overlooked, the maximal date range available in each database was used. Medical Subject Headings terms of “Pilates”, “Pilates method”, and “Low Back Pain” and synonyms for low back pain were inputted in the title, abstract, and as able, the keyword fields to identify relevant evidence (Table 1).

**Table 1:** Search Strategy.

Database	Date Range	Key Words	Fields
Cochrane Library	1800–2014	(low back pain OR dorsalgia OR *spin* pain OR backache OR lumbago) AND (pilates OR pilates method)	Title, Abstract or Keyword
Cumulative Index to Nursing and Allied Health Literature	1970–2014	(low back pain OR dorsalgia OR *spin* pain OR backache OR lumbago) AND (pilates OR pilates method)	Title, Abstract, or Word in Subject Heading
Medline-	1928–2014	(low back pain OR dorsalgia OR *spin* pain OR backache OR lumbago) AND (pilates OR pilates method)	Title, Abstract or Keyword
Physiotherapy Evidence Database	1928–2014	low back pain AND pilates	Title and Abstract
Proquest (Dissertations and Theses, Medical and Health Complete, Nursing and Allied Health Source)	1928–2014	(low back pain OR dorsalgia OR *spin* pain OR backache OR lumbago) AND (pilates OR pilates method)	Title, Abstract, or Subject Heading
Scopus	1960–2014	(low back pain AND pilates) OR (dorsalgia AND pilates) OR (*spin* pain AND pilates) OR (backache AND pilates) OR (lumbago AND pilates)	Title, Abstract, or Keyword
Sport Discus	1975–2014	(low back pain OR dorsalgia OR *spin* pain OR backache OR lumbago) AND (pilates OR pilates method)	Title, Abstract, or Keyword
Web of Science	1977–2014	(low back pain OR dorsalgia OR *spin* pain OR backache OR lumbago) AND (pilates OR pilates method)	Topic or Title

Preliminary searching revealed that expanding the literature searches to include “exercise”, “motor control”, or “core stability” did not identify any additional Pilates specific exercise studies, nor did changing the Boolean operator to “or”. Removing “low back pain” also did not identify any additional studies. Once RCTs were selected for inclusion, their reference lists were searched for additional, relevant studies that met inclusion criterion [16]–[19], [24]–[29]. In addition, reference lists of previous systematic reviews of this topic were searched to ensure relevant studies were not missed [6], [7], [15]–[19].

### Selection of Evidence

Selection of relevant studies was based on the study's title and the abstract, and as required, the full document. Two independent reviewers selected the evidence according to the selection criteria (CW, BH). Any disagreements were resolved through discussion with a third reviewer (AB). To be considered in this systematic review, studies needed to:

1. Be published in the English language, as access to interpreters was not available.
2. Be published in full so that the methodological quality of the study could be assessed alongside results. Abstracts were excluded as they contained insufficient data to enable analysis of methodological quality [31].
3. Be RCTs to limit the risk of bias in findings regarding efficacy [26]. If studies reported that they were RCTs but did not describe the randomisation procedure they were included in this review. If, however, studies reported that they were RCTs, but described a pseudo-random technique of allocating participants to groups, such as alternative allocation, they were excluded from this review [27], [28].
4. Assess the effectiveness of Pilates exercise where the term “Pilates” was used to describe the type of prescribed exercise being investigated. Exercises described as “motor control” or “lumbar stabilisation” did not suffice for Pilates. This is because Pilates may include other features apart from motor control and lumbar stabilisation [9].
5. Include participants with CLBP, that is, localised pain in the lumbar region of more than 3 months in duration [1]. If studies only included participants with low back pain of less than 3 months duration, they were excluded. This is because people with CLBP respond differently to treatment compared to those with acute or subacute symptoms [32]. If studies included participants with acute or subacute low back pain and CLBP, the study was included as findings were still considered relevant.
6. Use outcome measures with appropriate psychometric qualities that evaluate pain and/or functional ability in people with CLBP [33]. For example, the Visual Analog Scale and Numerical Rating Scale for pain, and the Oswestry Disability Questionnaire and Roland Morris Disability Questionnaire for functional ability. RCTs with outcome measures for pain and/or functional ability that did not have sufficient validity, reliability, or responsiveness were excluded to avoid imprecise measurements of treatment effect [33].

### Appraisal of Evidence

The methodological quality of included RCTs was evaluated by two independent reviewers using the McMaster Critical Review Form for Quantitative Studies (CW, BH) [34]. This critical appraisal tool was utilised because it is comprehensive in assessing methodological quality of quantitative evidence [35]. This critical appraisal tool also has good inter-rater reliability [36]–[38]. To confirm the reliability of scoring in this review, the percentage agreement and kappa score between the two reviewers was calculated [39]. Any disagreements between reviewers were resolved through discussion with a third reviewer (AB).

The McMaster Critical Review Form for Quantitative Studies directs reviewers to consider 16 items of methodological quality relating to the study's purpose, literature review, design, sample, outcomes, intervention, results and conclusions [34]. Guidelines for the appraisal of evidence in this review were created to assist reviewers in consistently evaluating methodological quality (Table 2). These were based on guidelines provided by the authors of the McMaster appraisal tool [34], [38].

**Table 2:** Modified Guidelines for use of the McMasters Critical Appraisal Form for Quantitative Studies^34^.

Item	Essential Criteria
1. Purpose	Do the authors clearly state that the aim of the study, which is to evaluate the effect of Pilates exercise in individuals diagnosed with chronic low back pain (CLBP)?
2. Literature review	Do the authors justify, by identifying gaps in the literature, the need to undertake further research into the effectiveness of Pilates exercise for individuals diagnosed with CLBP?
3. Study design	Have the authors used a randomised controlled trial to answer study aims, that is, to evaluate the effectiveness of Pilates exercise in people with CLBP?
4. Blinding	Have the authors used assessor blinding to minimise bias?
5. Sample description	Have the authors described the sample in terms of age, gender, and at least one measure of disability due to CLBP?
6. Sample size	Have the authors justified their sample size through a power calculation or post hoc analysis (and recruited sufficient numbers)?
7. Ethics and consent	Have the authors documented ethical approval for the research and gained informed consent by participants?
8. Validity of outcomes	Did the authors use outcome measures that are valid for use in people with CLBP to assess all outcome variables?
9. Reliability of outcomes	Did the authors use outcome measures that are reliable for use in people with CLBP to assess all outcome variables?
10. Intervention description	Did the authors provide sufficient information to enable reproduction of the intervention?
11. Statistical significance	Did the authors report the results for at least one outcome measure in line with study aim and in terms of statistical significance?
12. Statistical analysis	Did the authors use appropriate statistical analyses in evaluating results according to their aim?
13. Clinical importance	Did the authors reflect on the clinical importance of results for people diagnosed with CLBP?
14. Conclusions	Did the authors provide appropriate conclusions considering the study method and results?
15. Clinical implications	Did the authors discuss clinical implications of the results in terms of treatment of CLBP and in directing further research?
16. Study limitations	Did the authors identify limitations of the study methodology and results?

If RCTs met each criterion outlined in the appraisal guidelines, they received a score of “one” for that item, or, if they did not meet the criteria, they received a score of “zero”. Individual item scores were then summated to provide a total score of methodological quality out of 16, with higher scores reflecting greater methodological quality. Once quality scores were calculated, these were divided into five qualitative categories of poor (score  = 0–8), fair (score  = 9–10), good (score  = 11–12), very good (score  = 13–14) and excellent (score = 15–16) methodological quality, as defined in previous research [38].

### Data Extraction and Syntheses

The number of included RCTs and their methodological quality were summarised using descriptive statistics. The author(s), year of publication, and details regarding participants, interventions, comparison treatments, outcome measures, were extracted from RCTs by the primary author (CW), and tabulated. To determine whether a meta-analysis of study findings could be performed, the clinical and statistical heterogeneity of RCTs was assessed [15], [40]–[41].

Clinical heterogeneity of RCTs was assessed by comparing differences in the population, intervention, comparison treatments, outcome measures, and timing of reassessment of individual studies [15]. Statistical heterogeneity was assessed by calculation of an i^2^ statistic of studies with similar comparison treatment groups using the Cochrane Review Manager (version 5.2) software [41]–[43]. If i^2^ was greater than 75%, studies were considered to have substantial heterogeneity [41]. If substantial clinical and statistical heterogeneity were present, pooling results in a meta-analysis was deemed inappropriate.

Key findings of RCTs were expressed in terms of between-group mean differences and 95% confidence intervals. If between-group mean differences and 95% confidence intervals were not provided by RCTs, these were calculated from post-treatment mean values and standard deviations using the Physiotherapy Evidence Database calculator [44]. For the randomised cross-over trial, the between-group mean difference and 95% confidence interval was calculated from the first comparison time period between the Pilates exercise and control group, as the carryover effect in relation to time of treatment and pain intensity was statistically significant [21].

Results for each outcome measures were considered to be statistically significant if the 95% confidence interval of the between group difference did not cross “zero” [45]. If a 95% confidence interval was unable to be calculated from results given, a p value less than 0.05 for the between group comparison was considered to indicate statistical significance [45]. Results were considered to be clinically significant in this review if the mean between-group difference was greater than the minimal clinically important difference reported in the literature [46], [47].

## Results

### Search Results

**Figure 1:**
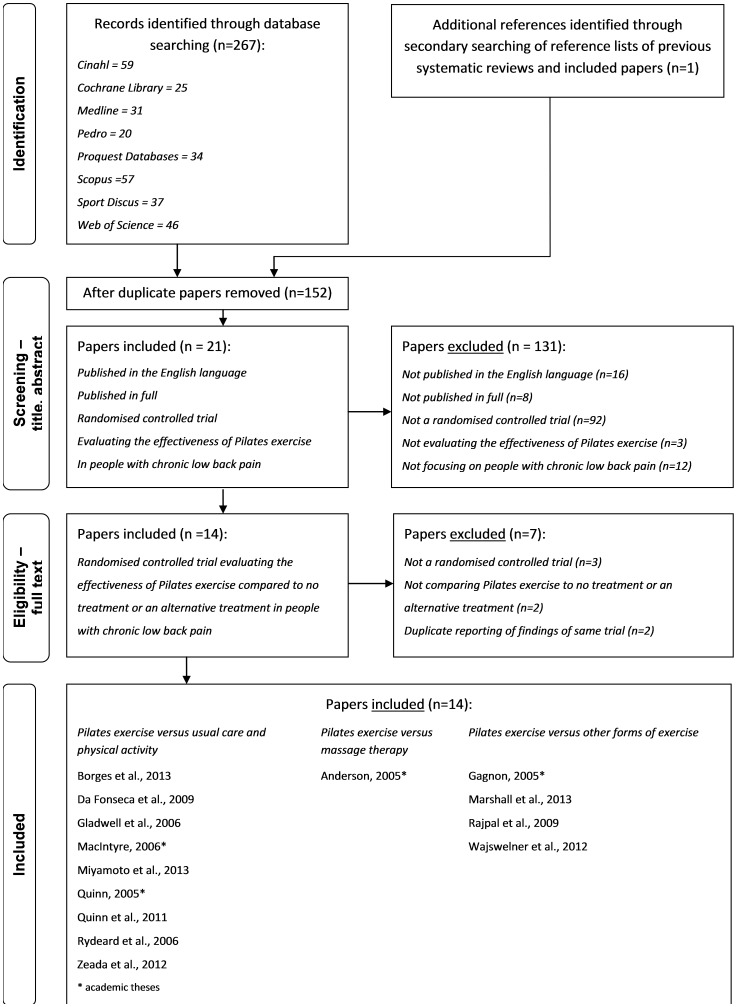
Results of Literature Search.

A total of 267 “hits” were obtained with database searching, and an additional RCT was identified when reviewing reference lists of previous systematic reviews (Figure 1) [48]. The majority of studies identified by this search strategy were excluded due to being duplicates (n = 115) or not being an RCT (n = 95). Other studies were excluded as they were not published in the English language (n = 16), did not assess the effectiveness of Pilates exercise in people with CLBP compared to other treatment (n = 17), were published in an abstract format (n = 8).

There was 100% agreement between the 2 reviewers regarding the 14 RCTs included in this review (Figure 1) [21], [23]–[26], [48]–[56]. Four RCTs were described in academic theses [48], [49], [51], [53] and 10 were published in academic journals [21], [23]–[26], [50], [52], [54]–[56]. It should be noted that 2 RCTs were reported across 2 papers but only 1 paper was included in this review to avoid duplication of findings [22], [23], [55], [57]. For one RCT, the paper that was published in a peer-reviewed journal was selected over the thesis to extract results [55], [57]. For the other RCT, the paper reporting on changes in pain and functional ability in the short and long term was included [23], rather than the paper reporting on outcomes only in the short term [22].

### Methodological Quality

There was 95% agreement between the two reviewers regarding item scores of the McMaster Critical Review Form for Quantitative Studies. This represents “almost perfect” inter-rater reliability (kappa score  = 0.88, p = 0.00) [39]. Discussion with the third reviewer was required to reach consensus regarding the adequacy of the description of Pilates exercise [27], presence of assessor bias [21], documentation of informed consent and ethical approval [51], use of valid and reliable outcome measures [51], and discussion of the statistical significance and clinical importance of results [48], [49]. One of the RCTs was published by an author of this review (PM) [23]. To avoid bias, this author was not involved in the stages of review of this RCT [23].

The methodological quality of studies ranged from 4 to 16, representing “poor” to “excellent” methodological quality (Table 3). RCTs published in the past 2 years were generally of higher quality, as were those published in journals compared to academic theses. According to the McMaster Quantitative Review Form, strengths in the methodological quality of most RCTs related to the provision of a clear purpose (Item 1), description of participants (Item 5), and documentation of ethic approval and consent (Item 7) [34]. The majority of RCTs also provided results in terms of statistical significance (Item 11) and conducted appropriate statistical analyses (Item 12) [34].

**Table 3:** Methodological Quality of Included Studies - McMaster Critical Review Form for Quantitative Studies [34].

Study	Individual Item																Total (/16)	Qualitative Descriptor [38]
	1	2	3	4	5	6	7	8	9	10	11	12	13	14	15	16		
*Pilates exercise versus usual care and physical activity*																		
1. Borges et al., 2013 [21]	1	1	1	1	1	1	1	0	0	1	1	1	0	1	1	1	**13**	Very good
2. da Fonseca et al., 2009 [50]	1	1	0	0	1	0	1	0	0	0	0	0	0	0	0	0	**4**	Poor
3. Gladwell et al., 2006 [52]	1	1	0	1	1	0	1	1	1	1	1	1	0	1	1	1	**13**	Very Good
4. MacIntyre, 2006 [48]	1	1	1	1	1	1	1	1	1	1	1	1	0	1	1	1	**15**	Excellent
5. Miyamoto et al., 2013 [24]	1	1	1	1	1	1	1	1	1	1	1	1	1	1	1	1	**16**	Excellent
6. Quinn, 2005 [53]	1	1	1	0	0	0	1	0	0	0	1	1	0	0	0	0	**6**	Poor
7. Quinn et al., 2011 [25]	1	1	1	1	1	0	1	0	1	1	1	1	1	1	1	0	**13**	Very good
8. Rydeard et al., 2006 [55]	1	1	1	1	1	0	1	1	1	0	1	1	1	1	1	1	**14**	Very good
9. Zeada et al., 2012 [56]	1	0	0	0	1	0	0	0	0	1	1	0	0	0	0	0	**4**	Poor
*Pilates exercise versus massage or other forms of exercise*																		
10. Anderson, 2005 [49]	1	1	1	1	1	0	0	0	0	1	0	1	0	1	1	1	**10**	Fair
11. Gagnon, 2005 [51]	1	1	1	0	1	0	1	0	0	0	1	1	0	1	1	1	**10**	Fair
12. Marshall et al., 2013 [23]	1	1	1	1	1	1	1	1	1	0	1	1	1	1	1	1	**15**	Excellent
13. Rajpal et al., 2009 [54]	0	0	0	0	0	0	1	0	0	1	1	1	0	0	0	1	**5**	Poor
14. Wajswelner et al., 2012 [26]	1	1	1	1	1	1	1	1	1	1	1	1	1	1	1	1	**16**	Excellent

Several RCTs, however, did not ensure assessor blinding (item 4), recruit an adequate sample size (Item 6), or document the validity and/or reliability of outcome measures (Item 8, 9) [34]. Other RCTs did not provide adequate detail of Pilates exercise programs for replication (Item 10), or discuss the clinical importance of results (Item 14) (Table 3) [34].

### Description of Included Studies

A summary of the population, intervention, comparison, and outcome measures for each RCT is provided in Table 4 and 5. The number of participants per RCT ranged from 12 to 83, while the mean age of participants across RCTs ranged between 21 to 49 years of age. The ratio of female to male participants ranged from 5∶1 through to 1∶1, except in one study where only females were recruited [54].

**Table 4:** Description of Included Studies- Pilates exercise versus usual care and physical activity.

Study	Population	Intervention and Comparison	Outcome Measures [Timing]
1. Borges et al., 2013 [21]	22 participants with chronic low back pain (CLBP) and Human T-Lymphotrophic Virus	Pilates: 2 X 60 minute supervised sessions per week for 15 weeks; Equipment = Mat, Cadillac, Reformer; Supervision Ratio = ?11 clients: 1 therapist; Standardised protocol	Short Form – 36 - Pain
	Gender (Female: Male) = 2.7: 1.0	No Pilates: no change in daily activities for 15 weeks	Visual Analog Scale - Pain
	Age & (years) = 48.3 (10.0)		[0, 15 weeks]
	Baseline Pain Intensity & (/10) a = Pilates 7.2 (2.4); Comparison 6.9 (2.5)		
2. da Fonseca et al., 2009 [50]	17 people with CLBP	Pilates: 2 sessions per week for 15 sessions; Equipment = Mat	Visual Analog Scale – Pain
	Gender (Female: Male) = 2.4: 1.0	No Pilates: continue usual physical activity but no treatment apart from medications	[0, 7–8 weeks]
	Age& (years) = 33.1(11.6)		
	Baseline Pain Intensity& (/10)^a^ = Pilates 5.9 (2.0); Comparison 6.1 (1.8)		
3. Gladwell et al., 2006 [52]	34 people with non-specific CLBP	Pilates: 60 minutes, 1 X per week for 6 weeks (as well as home exercises); Equipment: Mat	Oswestry Disability Questionnaire
	Gender (Female: Male) = 4∶1	No Pilates: usual physical activity, no treatment apart from medication	Visual Analog Scale – Pain
	Age & (years) = 40.6(9.7)		[0, 6 weeks]
	Baseline Pain Duration & (years) = 10.4 (10.1)		
4. MacIntyre, 2006 [48]	32 participants with CLBP	Pilates: 1 X 60 minute supervised mat session per week and 3 X 10 minute home exercises sessions per week for 12 weeks; General protocol	Roland Morris Disability Questionnaire
	Gender (Female: Male) Pilates = 3.0: 1.0; Comparison = 4.3: 1.0	No Pilates	Visual Analog Scale – Pain
	Age & (years) Pilates = 33.2 (7.7); Comparison = 46.7 (14.4)	(Both groups could undertake physiotherapy and exercise as required)	[0, 3, 12 weeks]
	Baseline Pain Intensity & (/10) a = Pilates 5.1 (2.0); Comparison 4.8 (1.7)		
	Baseline Disability & (/24) t = Pilates 7.0 (3.1); Comparison 7.4 (3.4)		
5. Miyamoto et al., 2013 [24]	86 participants with non-specific CLBP	Pilates: 2 X 60 minute supervised mat sessions per week for 6 weeks with education; Supervision ratio = 1∶1; General protocol but graded to individual ability	Numeric Rating Scale (11 point) - Pain
	Gender (Female: Male) = Pilates 5.0: 1.0; Comparison 3.8: 1.0	No Pilates: Education booklet and physiotherapy advice 2X per week for 6 weeks (Both groups could take medication as required)	Roland Morris Disability Questionnaire
	Age & (years) = Pilates 40.7 (11.8); Comparison 38.3 (11.4)		[0, 6, 24 weeks]
	Baseline Pain Intensity & (/10) + = Pilates 6.6 (1.5); Comparison 6.5 (1.7)		
	Baseline Disability & (/24) t = Pilates 9.7 (4.5); Comparison 10.5 (5.4)		
6. Quinn, 2005 [53]	22 participants with CLBP	Pilates: 2 X 45–60 minute supervised mat sessions per week for 12 weeks; Standardised protocol	Oswestry Disability Questionnaire
	Age & (years) = Pilates 46.3 (6.7) years; Comparison 34.7 (7.3)	No Pilates: usual daily activities for 12 weeks, and no new exercise program	[0, 12 weeks]
	Baseline Disability & (/100)^s^ = Pilates 25.9 (10.7); Comparison 22.0 (8.7)		
7. Quinn et al., 2011 [25]	29 participants with CLBP who had undergone physiotherapy treatment but had poor core stability and residual pain	Pilates: 1 X 60 minute supervised mat sessions and 5 X 15 minute home exercises per week for 8 weeks; Supervision ratio = 3–6:1; Standardised protocol but modified as required	Roland Morris Disability Questionnaire
	Age & (years) = Pilates 41.8 (13.8); Comparison 44.1 (12.5)	No Pilates: (or further treatment) for 8 weeks	Visual Analog Scale– Pain
	Baseline Pain Intensity & (/100) ∧ = Pilates 40.4 (14.6); Comparison 39.9 (19.9)		[0, 8 weeks]
	Baseline Disability & (/24) t = Pilates 6.9 (4.6); Comparison 7.7 (5.0)		
8. Rydeard et al., 2006 [55]	39 physically active participants with subacute, recurrent, or chronic low back pain	Pilates: 3 X 60 minute supervised sessions and 6 X 15 minute home exercises per week for 4 weeks; Equipment: Mat, Reformer; Standardised protocol	Numerical Rating Scale (101 point) – Pain
	Gender (Female: Male) = Pilates 2.0: 1.0; Comparison 1.6: 1.0	No Pilates: continued regular activity and consultation with medical and health care professionals	Roland Morris Disability Questionnaire - Hong Kong
	Age & (years) = Pilates 37.0 (9.0); Comparison 34.0 (8.0)		[0, 4 weeks]
	Baseline Pain Intensity & (/100) b = Pilates 23.0 (17.7); Comparison 30.4 (17.6)		
	Baseline Disability & (/24) t = Pilates 3.1(2.5); Comparison 4.2 (3.6)		
9. Zeada, 2012 [56]	20 athletes with chronic low back pain	Pilates: 4 sessions per week for 8 weeks; Equipment: Mat; Standardised protocol	Roland Morris Disability Questionnaire
	Age & (years) = Pilates 23.5 (2.4); Comparison 26.2(3.6)	No Pilates: 8 weeks	[0, 8 weeks]
	Baseline Disability & (/24) t = Pilates 7.4(1.2); Comparison 6.5 (0.9)		

& values represent Mean [Standard Deviation];

a as measured by Visual Analog Scale (11 point);

t as measured by Roland Morris Disability Questionnaire;

+ as measured by Numerical Rating Scale (11 point);

$ as measured by Oswestry Disability Index;

∧ as measured by Visual Analog Scale in mm;

b as measured by the Numerical Rating Scale (101 point).

**Table 5:** Description of Included Studies- Pilates exercise versus massage or other forms of exercise.

Study	Population	Intervention and Comparison	Outcome Measures [Timing]
1. Anderson, 2005 [49]	21 people with chronic or recurrent low back pain	Pilates: 2 X 50 minute supervised sessions per week for 6 weeks; Equipment = Reformer; Standardised protocol	Miami Back Index – Pain and Disability
	Gender (Female: Male) = 0.9: 1.0	Massage: 2 X 30 minute sessions per week for 6 weeks	Oswestry Disability Questionnaire
	Age & (years) = Pilates 42.4 (12.0); Comparison 44.0 (13.7)		[0, 6 weeks]
	Baseline Pain Intensity & (/10) # = Pilates 6.4 (2.5); Comparison 7.3 (1.7)		
	Baseline Disability & (/100)^s^ = Pilates 18.6 (5.9); Comparison 16.7 (4.2)		
2. Marshall et al., 2013 [23]	64 participants with CLBP	Pilates: 3 X 50–60 minute supervised sessions per week for 8 weeks; Equipment = Mat, Reformer; Supervision Ratio = 10 clients: 1 therapist; Standardised protocol	Oswestry Disability Questionnaire
	Gender (Female: Male) = 1.7: 1.0	Cycling: 3 X 50–60 minutes supervised indoor stationary cycle training for 8 weeks	Visual Analog Scale – Pain
	Age & (years) 36.2(6.2)		[0, 8, 24 weeks]
	Baseline Pain Intensity & (/10) a = Pilates 3.6 (2.1); Comparison 4.5 (2.5)		
	Baseline Disability & (/100)^s^ = Pilates 25.4 (11.2); Comparison 24.0 (11.9)		
3. Gagnon, 2005 [51]	12 participants with acute and chronic low back pain	Pilates: 1–2 X 30–45 minute supervised mat sessions per week for 6–7 weeks; Standardised protocol	Revised Oswestry Disability Index
	Gender (Female: Male) = Pilates 5.0: 1.0; Comparison 2.0: 1.0	Traditional lumbar stabilisation exercise: 1–2 X 30–45 minute supervised mat sessions per week for 6–7 weeks	Visual Analog Scale – Pain
	Age & (years) = Pilates 36.0 (11.4); Comparison 30.3 (12.4)	(Both groups could continue physiotherapy treatment and home exercises as indicated)	[0, 4, 6–7 weeks]
	Baseline Pain Intensity & (/10) a = Pilates 3.9 (2.5); Comparison 2.1 (1.7)		
	Baseline Disability & (/100)^s^ = Pilates 17.2 (6.1); Exercise 15.8 (3.7)		
4. Rajpal et al., 2009 [59]	40 females 20–30 years old with postural CLBP	Pilates exercise: Daily home exercise (10 repetitions with 10 second hold) over 4 weeks – progressed from crook lying, 4 point kneeling and knee extension on fit ball	Visual Analog Scale – Pain
	Age (years): Mean = 21.8	McKenzie exercise: Daily postural correction exercises (15–20 repetitions, 3 X per day) in sitting and standing	[0, 4 weeks]
5. Wajswelner et al., 2012 [26]	83 participants with CLBP or stiffness	Pilates: 2 X 60 minute supervised sessions per week, and daily home exercises for 6 weeks; Equipment: Mat, Reformer, Trapeze Table; Supervision Ratio: 4 clients: 1 therapist; Individualised, based on directional preferences	Numerical Rating Scale (11 point) - Pain
	Gender (Female: Male) = Pilates 1.3: 1.0; Comparison 1.2: 1.0	General exercise: 2 X 60 minute supervised sessions per week (including aerobic, stretching, strengthening, and stabilisation exercise) and daily home exercises for 6 weeks; Supervision Ratio: 4 clients: 1 therapist	Quebec Scale - Pain and Disability
	Age & (years) = Pilates 49.3 (14.1); Comparison 48.9(16.4)	(Both groups could utilise analgesic medication as required but no other form of treatment)	[0, 6, 12, 24 weeks]
	Baseline Pain Intensity & (/10) + = Pilates 4.9 (1.6); Comparison 4.6 (1.8)		
	Baseline Disability & (/100) t = Pilates 28.1 (11.4); Comparison 23.9 (14.0)		

& values represent Mean [Standard Deviation];

# as per Miami Back Index;

$ as measured by Oswestry Disability Index;

a as measured by Visual Analog Scale (11 point);

+ as measured by Numerical Rating Scale (11 point);

t as measured by Roland Morris Disability Questionnaire.

In terms of the Pilates interventions, most RCTs described supervised exercise programs delivered in 30 to 60-minute sessions, 1–3 times per week, for 4–15 weeks (Table 4, 5). Home exercises were incorporated in 6 RCTs as part of the Pilates exercise intervention [25], [26], [48], [52], [54], [55]. The supervision ratios of clients per therapist for supervised sessions ranged from 11∶1 to 1∶1, although not all RCTs provided this information. Use of specialised Pilates exercise equipment, such as a Reformer, was reported in 5 RCTs [21], [23], [26], [49], [55].

Pilates exercise was compared to usual care and physical activity in 9 RCTs, massage therapy in 1 RCT, and other forms of exercise in 4 RCTs (Table 4, 5). Usual care and physical activity could involve unknown other treatments [53], [56], no treatment [25], [50], [52], education [24], medications [24], [48], [55], or consultations with health professionals, such as physiotherapists [48], [55]. Other forms of exercise ranged from cycling [23], McKenzie exercise [54], traditional lumbar stabilisation exercise [51], and a mixed form of exercise including stretching, strengthening and stabilisation [26].

Variable outcome measures were used to investigate the effectiveness of Pilates exercise in reducing pain and improving functional ability in people with CLBP (Table 4, 5). These included the Visual Analog Scale and Numerical Rating Scale (11 and 101 point scales) for pain, and the Roland Morris Disability Questionnaire, Oswestry Disability Questions, Quebec Score, and the Miami Back Index for functional ability [33], [47]. Treatment outcomes were measured at different time periods, ranging from 4 to 24 weeks.

### Heterogeneity of Included Studies

Significant clinical heterogeneity was noted across RCTs in terms of the study population, intervention, and outcome assessment (Table 4, 5). Though all RCTs studied people with CLBP, some included people with acute and subacute symptoms, or other diagnoses [21], [49], [55]. Pilates exercise interventions also varied in terms of the duration of the intervention, level of supervision, incorporation of home exercises, education, and use of specialised equipment. Outcomes were also measured with different outcome measures at different time periods.

Comparison treatments also varied considerably across RCTs (Table 4, 5). When Pilates exercise was compared to usual care and physical activity, participants could access variable therapies depending on the RCT [24], [48], [55]. Usual physical activity also varied with some studies focusing on participants who were highly active [55], [56], while the others did not. When Pilates exercise was compared to other forms of exercise, comparison exercise regimes were either similar to Pilates exercise [26], [51], or quite distinct [23]. Also, in some studies, participants could access other interventions as well [26], [51].

Significant statistical heterogeneity was also observed when comparing outcomes achieved with Pilates exercise versus usual care and physical activity for pain (i^2^ = 90%, p<0.001), and functional ability (i^2^ = 87%, p<0.00001) between 4 and 12 weeks. Similarly, significant statistical heterogeneity was noted when comparing pain relief achieved with Pilates exercise versus other forms of exercise (i^2^ = 83%, p = 0.0006) between 4 and 8 weeks. Pooling individual study findings in a meta-analysis, then, was deemed inappropriate for these variables in the short term, given the clinical and statistical heterogeneity of RCTs [40], [41].

Moderate statistical heterogeneity was noted when comparing improvements in functional ability with Pilates exercise and other forms of exercise in the short term (4–8 weeks) (i^2^ = 44%, p = 0.17). At 24 weeks, mild statistical heterogeneity was evident when Pilates exercise was compared with other forms of exercise across the 2 RCTs for pain (i^2^ = 25%, p = 0.25) and functional ability (i^2^ = 9%, p = 0.29) [23], [26]. Nevertheless, the clinical heterogeneity of RCTs comparing Pilates exercise to other forms of exercise suggested a meta-analysis would be of limited benefit [40], [41].

### Findings of Included Studies

#### Pilates exercise versus usual care and physical activity

Four high quality RCTs reported a statistically significant difference in pain relief with Pilates exercise in the short term (4–15 weeks) [21], [24], [25], [55]. Two high quality RCTs, and one poor quality RCT, however, disagreed with these findings [48], [50], [52]. At 24 weeks, no statistically significance difference in pain relief with Pilates exercise and education versus education alone was reported (Table 6) [24].

**Table 6:** Effectivenessof Pilates exercise versus usual care and physical activity in reducing pain in people with chronic low back pain.

Study	Methodological Quality [Score]	Population [Sample size]	Intervention and Comparison	Outcome Measure(s)	Assessment Timing	Mean Difference [95% confidence interval]
1. Borges et al., 2013 [21]	Very good [13/16]	Chronic low back pain [n = 64]	Pilates exercise versus no change in physical activity	Visual Analog Scale	15 weeks	−4.1 [−6.3 to −1.8]^a^
2. da Fonseca et al., 2009 [50]	Poor [4/16]	Chronic low back pain [n = 17]	Pilates exercise versus no Pilates exercise	Visual Analog Scale	7–8 weeks	−1.9 [−5.0 to 1.2]
3. Gladwell et al., 2006 [52]	Very good [13/16]	Chronic low back pain [n = 34]	Pilates exercise versus usual care and physical activity	Visual Analog Scale – Present Pain	6 weeks	−0.2 [−0.8 to 0.4]
				Visual Analog Scale - Pain Diary	6 weeks	−0.3 [−0.9 to 0.3]+
4. MacIntyre, 2006 [48]	Excellent [15/16]	Non-specific chronic low back pain [n = 86]	Pilates exercise versus no change in physical activity^s^	Visual Analog Scale	3 weeks	−0.4 [−1.7 to 0.9]
					12 weeks	−1.6 [−3.2 to 0.0]
5. Miyamoto et al., 2013 [24]	Excellent [16/16]	Chronic low back pain for more than 6 months [n = 22]	Pilates exercise and education versus education alone	Numerical Rating Scale (11 point)	6 weeks	−2.2 [−3.2 to −1.1]^a^
					24 weeks	−0.9 [−1.9 to 0.1]
6. Quinn et al., 2011 [25]	Very good [14/16]	Chronic low back pain after physiotherapy [n = 29]	Pilates exercise versus no Pilates exercise	Visual Analog Scale	8 weeks	−1.5 [−2.1 to −0.9]^a^
7. Rydeard et al., 2006 [55]	Very good [14/16]	Subacute, chronic, or recurrent low back pain, physically active [n = 39]	Pilates exercise versus no change in physical activity^s^	Numerical Rating Scale (101 point)	4 weeks	−15.6 [−17.8 to −13.4]^a^

a statistically significant between group difference;

+ reported as statistically significant in study, but not calculated in this review;

s with or without usual care.

The statistically significant improvements in pain reported in the short term were clinically significant in 3 out of 4 RCTs [21], [24], [25]. This is because mean difference scores exceeded the minimal clinically important difference for their respective outcome measures. For example, Borges et al. (2013) and Quinn, Barry, and Barry (2011) described a mean reduction (and 95% confidence interval) on the Visual Analog Scale of 4.1 (1.8 to 6.3) and 1.5 (0.9 to 2.1) points respectively [21], [25]. The minimal clinically important difference for the Visual Analog Scale in people with CLBP has been reported in the literature as between 1.5 to 2 points [46], [47].

Similarly, Miyamoto, Costa, Glavanin, and Cabral (2013) reported a mean reduction (and 95% confidence interval) of pain on the 11 point Numerical Rating Scale of 2.2 (1.1 to 3.2) points where the minimal clinically important difference was 2 points [47]. Meanwhile, Rydeard, Leger, and Smith (2006) did not report a clinically significant improvement in pain, that is a mean reduction (and 95% confidence interval) of 15.6 (13.4 to 17.8) points on the 101 point Numerical Rating Scale [55]. This is lower than the estimated minimal clinically important difference of 20 points [46].

With regards to functional ability, 3 high quality RCTs reported statistically significant improvements with Pilates exercise in the short term (4–12 weeks) [24], [48], [55]. In contrast, 2 high quality RCTs and 3 poor quality RCTs did not [25], [50], [52], [53], [56]. Meanwhile, at 24 weeks, no statistically significance difference in improvement in functional ability with Pilates exercise with education versus education alone was reported (Table 7) [24].

**Table 7:** Effectiveness of Pilates exercise compared to usual care and physical activity in improving functional ability in people with chronic low back pain.

Study	Methodological Quality [Score]	Population [Sample size]	Intervention and Comparison	Outcome Measure(s)	Assessment Timing	Mean Difference [95% confidence interval]
1. Gladwell et al., 2006 [52]	Very good [13/16]	Chronic low back pain [n = 34]	Pilates exercise versus usual care and physical activity	Oswestry Disability Questionnaire	6 weeks	0.0 [−8.5 to 8.5]
2. MacIntyre, 2006 [48]	Excellent [15/16]	Non-specific chronic low back pain [n = 86]	Pilates exercise versus no change in physical activity^s^	Roland Morris Disability Questionnaire	3 weeks	−0.6 [−2.6 to 1.5]
					12 weeks	−2.6 [−5.2 to −0.1]^a^
3. Miyamoto et al., 2013 [24]	Excellent [16/16]	Chronic low back pain for greater than 6 months [n = 22]	Pilates exercise and education versus education alone	Roland Morris Disability Questionnaire	6 weeks	−2.7 [−4.4 to −1.0]^a^
					24 weeks	−1.4 [−3.1 to 0.0]
4. Quinn, 2005 [53]	Poor [6/16]	Chronic low back pain [n = 22]	Pilates exercise versus usual physical activity	Oswestry Disability Questionnaire	12 weeks	−7.1 [−17.6 to 3.4]
5. Quinn et al., 2011 [25]	Very good [14/16]	Chronic low back pain after physiotherapy [n = 29]	Pilates exercise versus no Pilates exercise	Oswestry Disability Questionnaire	8 weeks	1.3 [not given but p>0.05]
6. Rydeard et al., 2006 [55]	Very good [14/16]	Subacute, chronic, or recurrent low back pain, physically active [n = 39]	Pilates exercise versus no change in physical activity^s^	Oswestry Disability Questionnaire	4 weeks	−1.2 [−1.4 to −1.0]^a^
7. Zeada et al., 2012 [56]	Poor [4/16]	Athletes with chronic low back pain [n = 20]	Pilates exercise versus no Pilates exercise	Roland Morris Disability Questionnaire	8 weeks	1.7 [−0.4 to 3.8]+

s with or without usual care;

a statistically significant between group difference;

+ reported as statistically significant in the study.

Statistically significant short-term improvements in functional ability with Pilates exercise were not clinically significant. For example, MacIntyre (2005) and Miyamoto et al. (2013) reported a mean improvement (and 95% confidence interval) of 2.6 (1.5 to 5.2) and 2.7 (1.0 to 4.4) on the Roland Morris Disability Questionnaire respectively. These changes were less than the minimal clinically important distance of 3.5 to 5 points [46], [47]. Similarly, Rydeard et al. (2006) reported a mean change (and 95% confidence interval) of 1.2 (1.1 to 1.4) points on the Oswestry Disability Questionnaire which is below the minimal clinically important difference of 10 points [46], [47].

#### Pilates exercise versus massage therapy

Only one RCT of fair quality compared the effectiveness of Pilates exercise to massage therapy [49]. This RCT did not report any statistically significant differences in pain or functional ability between groups at 6 weeks (Table 8, 9). As a consequence, there were no clinically significant differences noted between Pilates exercise and massage therapy.

**Table 8:** Effectiveness of Pilates exercise versus massage or other forms of exercise in reducing pain in people with chronic low back pain.

Study	Methodological Quality [Score]	Population [Sample size]	Intervention and Comparison	Outcome Measure(s)	Assessment Timing	Mean Difference [95% confidence interval]
1. Anderson, 2005 [49]	Fair [10/16]	Chronic or recurrent low back pain [n = 21]	Pilates exercise versus massage	Miami Back Index (Pain)	6 weeks	−10.8 [−25.9 to 4.3]
2. Marshall et al., 2013[23]	Excellent [15/16]	Chronic low back pain [n = 64]	Pilates exercise versus stationary cycling	Visual Analog Scale (Current pain)	8 weeks	−1.1 [−2.1 to −0.1]^a^
					24 weeks	−1.4 [−2.6 to −0.2]
				Visual Analog Scale (Worst pain)	8 weeks	−0.4 [−1.4 to 0.6]
3. Gagnon, 2005 [51]	Fair [10/16]	Acute and chronic low back pain [n = 12]	Pilates exercise versus lumbar stabilisation	Visual Analog Scale	4 weeks	0.8 [−1.3 to 2.9]
					6–7 weeks	0.6 [−1.7 to 2.8]
4. Rajpal et al., 2009 [54]	Poor [5/16]	Females with chronic low back pain [n = 40]	Pilates exercise versus McKenzie exercise	Visual Analog Scale	4 weeks	−1.4 [−2.1 to −0.7]^a^^,^ $
5. Wajswelner et al., 2012 [26]	Excellent [16/16]	Chronic low back pain [n = 83]	Pilates exercise versus general exercise (mixed)	Numerical Rating Scale (11 point)	6 weeks	−0.5 [−1.3 to 0.3]
					12 weeks	−0.6 [−1.5 to 0.3]
					24 weeks	0.3 [−0.7 to 1.2]

a statistically significant between group difference;

$ based on comparison of pre and post treatment scores.

**Table 9:** Effectiveness of Pilates exercise versus massage or other forms of exercise in improving functional ability in people with chronic low back pain.

Study	Methodological Quality [Score]	Population [Sample size]	Intervention and Comparison	Outcome Measure(s)	Assessment Timing	Mean Difference [95% confidence interval]
1. Anderson, 2005 [49]	Fair [10/16]	Chronic or recurrent low back pain [n = 21]	Pilates exercise versus massage therapy	Miami Back Index (Disability)	6 weeks	−7.9 [−1.4 to 0.3]
				Oswestry Disability Questionnaire	6 weeks	−4.0 [−10.0 to 2.0]
2. Marshall et al., 2013 [23]	Excellent [15/16]	Acute and chronic low back pain [n = 12]	Pilates exercise versus stationary cycling	Oswestry Disability Index	8 weeks	−6.5 [−11.8 to −1.1]^a^
					24 weeks	4.4 [−0.7 to 9.5]
3. Gagnon, 2005 [51]	Fair [10/16]	Chronic low back pain [n = 32]	Pilates exercise versus lumbar stabilisation	Oswestry Disability Index	4 weeks	−3.0 [−11.1 to 5.1]
					6–7 weeks	−2.2 [−10.9 to 6.5]
4. Wajswelner et al., 2012 [26]	Excellent [16/16]	Chronic low back pain [n = 83]	Pilates exercise versus general exercise	Quebec Score	6 weeks	1.8 [−3.1 to 6.7]
					12 weeks	−0.8 [−6.4 to 4.8]
					24 weeks	−1.1 [−5.8 to 3.6]

a statistically significant between group difference.

#### Pilates exercise versus other forms of exercise

When Pilates exercise was compared to other forms of exercise, conflicting results in terms of pain relief were reported in the short term (4–8 weeks) [23], [26], [51], [54]. One high quality and low quality RCT reported statistically significant improvements [23], [54], while another high quality and low quality RCT did not [26], [51]. At 24 weeks, though, there was agreement in 2 high quality RCTs that Pilates exercise resulted in equivalent improvements in pain as other forms of exercise (Table 8) [23], [26].

The improvement in pain suggested in one high quality and low quality RCT in the short term was not clinically significant [23], [54]. For example, Marshall, Kennedy, Brooks, and Lonsdale (2013) reported a mean decrease (and 95% confidence interval) of 1.1 (0.1 to 2.1) points on the Visual Analog Scale, and Rajpal, Arora, and Chauhan (2009) reported a mean decrease (and 95% confidence interval) of 1.4 (0.7 to 2.1) points on the Visual Analog Scale [23], [54]. These scores were less than the minimal clinically important difference of 1.5–2 points on the Visual Analog Scale [46], [47].

With regards to functional ability, one high quality RCT reported a statistically significant improvement with Pilates exercise over other forms of exercise in the short term [23]. In contrast, another high quality and low quality RCT did not report a statistically significant difference in improvement [26], [51]. At 24 weeks, however, 2 high quality RCTs agreed that Pilates exercise offered similar improvements in functional ability as other forms of exercise (Table 9) [23], [26].

The statistically significant improvement in functional ability reported by Marshall et al. (2013) in the short term was not clinically significant [23]. This is because the mean improvement (and 95% confidence interval) in functional ability was 6.5 (1.1 to 11.8) points on the Oswestry Disability Index which is less than the minimal clinically important difference of 10 points [23], [46], [47].

## Discussion

This systematic review provides an update on the effectiveness of Pilates exercise in reducing pain and improving functional ability in people with CLBP based on current evidence. It provides a meta-synthesis of findings from 14 RCTs, including recently published RCTs that have not been included in other reviews [21], [23]–[26], [48]–[56]. A meta-analyses was not conducted due to the heterogeneity of RCTs [40], [41].

### Pilates exercise versus usual care and physical activity

Pilates exercise results in statistically significant improvements in pain and functional ability in the short term in people with CLBP. This conclusion is based on the balance of evidence, where more high quality RCTs have reported these findings [21], [24], [25], [55]. In addition, short term improvements in pain may be clinically significant, but not improvements in functional ability [45]–[47].

Another conclusion of this review is that superior improvements with Pilates exercise compared to usual care and physical activity is unlikely at 24 weeks. This is based on research evidence of one high quality RCT that has investigated the longer term effect of Pilates exercise [24]. In this RCT, though, participants had ceased Pilates exercises at 6 weeks, and it is not known if a longer lasting effect may have been present if the intervention was continued for more than 6 weeks, as is recommended in the literature [13].

These systematic review findings are similar to those of another review in that a statistically significant reduction in pain with Pilates exercise was achieved when compared to no Pilates exercise [17]. This current review clarifies, though, that this improvement may only be in the short term, and that this change may be clinically significant. In relation to functional ability, these review findings contrast with other systematic reviews as a statistically significant improvement in functional ability in the short term was identified [6], [7], [17]. This difference may be due to inappropriate meta-analyses in some reviews and variable grouping of comparison treatments [13]. The size of functional improvements in RCTs in this review, however, do not appear to be clinically significant [46], [47].

It should be acknowledged that not all RCTs in this review agreed regarding the effectiveness of Pilates exercise compared to usual care and physical activity [48], [50], [52], [53]. Different results may be explained by the variable methodological quality of RCTs. For example, the majority of high quality RCTs reported statistically significant findings (4/6), while lower quality RCTs did not (2/2) [50], [53].

Different results may also be due to small sample sizes or co-interventions within RCTs. Three of the 4 RCTs that did not find statistically significant findings were under-powered with small sample sizes, meaning that treatment changes may have been less easily detected [50], [52], [53]. The other RCT had a large sample size, but allowed the comparison group to access other interventions, such as physiotherapy and medications [48]. This may have led to the effectiveness of Pilates exercise being under estimated as the between group difference in outcome may have been reduced.

In addition, different RCT outcomes may have related to variable Pilates exercise regimes. For example, RCTs with statistically significant results prescribed supervised exercise sessions more than once a week, often with the use of specialised equipment [21], [24], [25], [55]. It is therefore recommended that clinicians replicate Pilates exercise programs contained within RCTs with statistically significant results to maximise treatment outcomes.

### Pilates exercise versus massage therapy

Only one RCT compared Pilates exercise to massage therapy [49]. No statistically significant difference in improvements in pain or functional ability was noted at 6 weeks. More high quality RCTs, though, are required to confirm these findings due to the “fair” methodological quality of this RCT [34], [38].

### Pilates exercise versus other forms of exercise

Based on current evidence, it is difficult to conclude on the short-term effectiveness of Pilates exercise in people with CLBP compared to other forms of exercise. Statistically significant improvements in pain and functional ability have been reported in one high quality RCT [23], but not in other high quality RCTs [26]. The clinical significance of reported statistically significant improvements is also unlikely [23], [46], [47]. There is consensus across high quality RCTs, though, that people with CLBP will experience equivalent improvements in pain and functional ability with Pilates exercise or alternative forms of exercise at 24 weeks [23], [26].

Authors of this review therefore suggest that Pilates exercise is unlikely to provide superior improvements in pain and functional ability compared to other forms of exercise, at least in the long term. Findings of this review are similar to those of previous systematic reviews in that improvements in pain and functional ability with Pilates exercise compared to other forms of exercise have not been reported as statistically significant [6], [7], [17]. This review is different, however, in that it acknowledges that there could be differences in the short term.

There are two reasons why authors of this review have not ruled out the possibility of Pilates exercise offering superior short-term benefit over other forms of exercise. First, one of the two RCTs that reported no difference in the short term was of “fair” methodological quality [34], [38]. This meant that findings were likely to be more biased than that of higher quality RCTs [38]. Second, comparison exercise treatments were variable and it could be possible that Pilates exercise is more effective than some types of exercise, but not others. When Pilates exercise was compared to a distinctly different form of exercise, cycling, there was a statistically significant difference in outcome [23]. When compared to exercises involving lumbar stabilisation, however, no difference was noted [26], [51]. Future research should investigate the relative effectiveness of Pilates exercise to different forms of exercise.

### Limitations

Limitations of this systematic review relate to the inclusion of only RCTs published in the English language and consequent language bias. Of the 16 studies excluded based on their language, however, only 2 appeared to be potentially relevant RCTs when reviewing titles and abstracts translated into the English language [58], [59]. Another limitation was the focus of this review on outcomes of pain and functional ability in people with CLBP. Other outcomes may have also been clinically important, such as quality of life [33], [60]. In addition, the methodological quality of RCTs was summarised by a total score out of 16 using the McMaster Quantitative Review Form criteria [34]. This approach can lead to oversimplification of methodological quality as all items of the scale are weighted evenly [61].

The strength of the review findings was also influenced by the availability and diversity of primary evidence. The limited number of RCTs that had compared Pilates exercise to massage therapy and other forms of exercise lessened the certainty of results [15]. The small sample sizes and short term follow up of many RCTs also affected the precision of findings [62]. Moreover, the heterogeneity of study populations, interventions, comparison treatments, outcome measures, and timing of reassessment prevented conduction of meaningful meta-analyses of RCTs [40], [41].

## Conclusion

According to this systematic review, Pilates exercise results in statistically significant improvements in pain and functional ability in the short term compared to usual care and physical activity in people with CLBP [21], [24], [25], [55]. Changes in pain are more likely to be clinically significant than improvements in functional ability. At 24 weeks, though, improvements with Pilates exercise and education may be equivalent to those achieved with education alone [24]. When Pilates exercise is compared to massage therapy or other forms of exercise, equivalent improvements in pain and functional ability have been reported in people with CLBP [23], [26], [49].

### Implications

This systematic review provides an update on the effectiveness of Pilates exercise in people with CLBP that may be used to assist clinical decision-making. Future research should investigate optimal Pilates exercise regimes for people with CLBP, including appropriate frequencies and length of programs, supervision ratios, use of home exercises, and specialised equipment [13]. Future RCTs should also investigate the long term efficacy of Pilates exercise to other treatments, such as massage, and confirm if there is any difference in effectiveness between Pilates exercise and various forms of exercise, such as aerobic exercise versus lumbar stabilisation [15]. Research into whether some people with CLBP may benefit from Pilates exercise more than others may also assist in clinical decision-making on whether Pilates exercise is suitable for individual clients [63], [64].

## Supporting Information

Checklist S1 (DOC)

## References

[1] Charlton JE (2005) Core Curriculum for Professional Education in Pain, 3^rd^ ed. Seattle: International Association of the Study of Pain Press.

[2] Dagenais S, Caro J, Haldeman S (2008) A systematic review of low back pain cost of illness studies in the United States and internationally. Spine J 8: 8–20. 18164449 10.1016/j.spinee.2007.10.005

[3] Hoy D, March L, Brooks P, Woolf A, Blyth F, et al (2010) Measuring the global burden of low back pain. Best Pract Res Clin Rheumatol 24: 155–165. 20227638 10.1016/j.berh.2009.11.002

[4] Woolf AD, Pfleger B (2003) Burden of major musculoskeletal conditions. Bull World Health Organ 81: 646–656. 14710506 PMC2572542

[5] Brennan S, French H (2008) A questionnaire survey of the knowledge and use of Pilates based exercise for chronic low back pain amongst Irish physiotherapists. Phys Ther Rev 13: 212–213.

[6] Aladro-Gonzalvo AR, Araya-Vargas GA, Machado-Diaz M, Salazar-Rojas W (2013) Pilates-based exercise for persistent, non-specific low back pain and associated functional disability: A meta-analysis with meta-regression. J Bodyw Mov Ther 17: 125–136. 23294694 10.1016/j.jbmt.2012.08.003

[7] Pereira LM, Obara K, Dias JM, Menacho MO, Guariglia DA, et al (2012) Comparing the Pilates method with no exercise or lumbar stabilization for pain and functionality in patients with chronic low back pain: Systematic review and meta-analysis. Clin Rehabil 26: 10–20. 21856719 10.1177/0269215511411113

[8] Latey P (2001) The Pilates method: History and philosophy. J Bodyw Mov Ther 5: 275–282.

[9] Wells C, Kolt GS, Bialocerkowski A (2012) Defining Pilates exercise: A systematic review. Complement Ther Med 20: 253–262. 22579438 10.1016/j.ctim.2012.02.005

[10] Hayden JA, van Tulder MW, Tomlinson G (2005) Systematic review: Strategies for using exercise therapy to improve outcomes in chronic low back pain. Ann Intern Med 142: 776–785. 15867410 10.7326/0003-4819-142-9-200505030-00014

[11] Macedo LG, Maher CG, Latimer J, McAuley JH (2009) Motor control exercise for persistent, nonspecific low back pain: A systematic review. Phys Ther 89: 9–25. 19056854 10.2522/ptj.20080103

[12] Pillastrini P, Gardenghi I, Bonetti F, Capra F, Guccione A, et al (2012) An updated overview of clinical guidelines for chronic low back pain management in primary care. Joint Bone Spine 79: 176–185. 21565540 10.1016/j.jbspin.2011.03.019

[13] Wells C, Kolt GS, Marshall P, Bialocerkowski A (2014) The definition and application of Pilates exercise to treat people with chronic low back pain: A Delphi survey of Australian physical therapists. Phys Ther 94, 10.2522/ptj.20130030 24179139

[14] Da Luz MA, Costa LOP, Fuhro FF, Manzoni ACT, Oliveira NTB, et al (2014) Effectiveness of mat Pilates or equipment-based Pialtes exercises in patiences with chronic nonspecific low back pain: A randomised controlled trial. Phys Ther 94: 623–631, 10.2522/ptj.20130277 24435105

[15] Wells C, Kolt GS, Marshall P, Hill B, Bialocerkowski A (2013) Effectiveness of Pilates exercise in treating people with chronic low back pain: A systematic review of systematic reviews. BMC Medical Research Methodology 13: 7 Available: http://www.biomedcentral.com/1471-2288/13/7. Accessed 4 February 2014 23331384 10.1186/1471-2288-13-7 PMC3563510

[16] La Touche R, Escalante K, Linares MT (2008) Treating non-specific chronic low back pain through the Pilates Method. J Bodyw Mov Ther 12: 364–370. 19083695 10.1016/j.jbmt.2007.11.004

[17] Lim ECW, Poh RLC, Low AY, Wong WP (2011) Effects of Pilates-based exercises on pain and disability in individuals with persistent non-specific low back pain: A systematic review with meta-analysis. J Orthop Sports Phys Ther 41: 70–80. 20972339 10.2519/jospt.2011.3393

[18] Miyamoto GC, Costa LO, Cabral CM (2013) Efficacy of the Pilates method for pain and disability in patients with chronic nonspecific low back pain: A systematic review with meta-analysis. Braz J Phys Ther 17: 517–532, 10.1590/S1413-35552012005000127 24346291 PMC4207151

[19] Posadzki P, Lizis P, Hagner-Derengowska M (2011) Pilates for low back pain: A systematic review. Complement Ther Clin Pract 17: 85–89. 21457897 10.1016/j.ctcp.2010.09.005

[20] Costa LOP, Hancock M, Maher C, Ostelo RWJG, Cabral CMN, et al (2012) Pilates for low back pain (Protocol). Cochrane Database of Systematic Reviews 12: CD010265 10.1002/14651858.CD010265 PMC8078578 26133923

[21] Borges J, Baptista AF, Santana N, Souza I, Kruschewsky RA, et al (2014) Pilates exercises improve low back pain and quality of life in patients with HTLV-1 virus: A randomised crossover clinical trial. J Bodywork and Movt Ther 18: 68–74. 10.1016/j.jbmt.2013.05.010 24411152

[22] Brooks C, Kennedy S, Marshall PW (2012) Specific trunk and general exercise elicit similar changes in anticipatory postural adjustments in patients with chronic low back pain: A randomized controlled trial. Spine 37: E1543–E1550. 22926279 10.1097/BRS.0b013e31826feac0

[23] Marshall PW, Kennedy S, Brooks C, Lonsdale C (2013) Pilates exercise or stationary cycling for chronic nonspecific low back pain: Does it matter? A randomised controlled trial with 6 month follow-up. Spine 38: E952–959. 23615384 10.1097/BRS.0b013e318297c1e5

[24] Miyamoto GC, Costa LOP, Glavanin T, Cabral CMN (2013) Efficacy of the addition of modified Pilates exercises to minimal intervention in patients with chronic low back pain: A randomised controlled trial. Phys Ther 93: 310–320. 10.2522/ptj.20120190 23064732

[25] Quinn K, Barry S, Barry L (2011) Do patients with chronic low back pain benefit from attending Pilates classes after completing conventional physiotherapy treatment? Physiotherapy Ireland 32: 5–12.

[26] Wajswelner H, Metcalf B, Bennell K (2012) Clinical pilates versus general exercise for chronic low back pain: Randomized trial. Med Sci Sports Exerc 44: 1197–1205. 22246216 10.1249/MSS.0b013e318248f665

[27] Kunz R, Vist GE, Oxman AD (2007) Randomisation to protect against selection bias in healthcare trials. Cochrane Database Syst Rev 2 : Article Number MR000012Article Number MR000012. 10.1002/14651858.MR000012.pub2 17443633

[28] National Health and Medical Research Council (NHMRC) (2009) NHMRC additional levels of evidence and grades for recommendations for developers of guidelines. Canberra: National Health and Medical Research Council.

[29] Collins J, Fauser B, Bart CJM (20050 Balancing the strengths of systematic and narrative reviews. Hum Reprod Update 11: 103–104. 15618290 10.1093/humupd/dmh058

[30] Cook D, Mulrow C, Haynes R (1997) Systematic reviews: Synthesis of best evidence for clinical decisions. Ann Intern Med 126: 376–380. 9054282 10.7326/0003-4819-126-5-199703010-00006

[31] Hopewell S, Clarke M, Moher D, Wager E, Middleton P, et al (2008) CONSORT for reporting randomised trials in journal and conference abstracts. Lancet 371: 281–283. 18221781 10.1016/S0140-6736(07)61835-2

[32] Hayden JA, van Tulder MW, Malmivaara A, Koes BW (2005) Exercise therapy for treatment of non-specific low back pain. Cochrane Database Syst Rev 3 : Article Number CD000335. 10.1002/14651858.CD000335.pub2 PMC10068907 16034851

[33] Chapman JR, Norvell DC, Hermsmeyer JT, Bransford RJ, DeVine J, et al (2011) Evaluating common outcomes for measuring treatment success for chronic low back pain. Spine 36: S54–68. 21952190 10.1097/BRS.0b013e31822ef74d

[34] Law M, MacDermid J (1998) Evidence-based rehabilitation (2^nd^ Ed.). Thorofare, New Jersey: Slack.

[35] Katrack P, Bialocerkowski AE, Massy-Westropp N, Kumar VSS, Grimmer KA (2004) A systematic review of the content of critical appraisal tools. BMC Med Res Methodol 4:22 . Available: http://www.biomedcentral.com/1471-2288/4/22. Accessed 4 February 2014. 10.1186/1471-2288-4-22 PMC521688 15369598

[36] Lekkas P, Larson T, Kumar S, Grimmer K, Nyland L, et al (2007) No model of clinical education for physiotherapy students is superior to another: A systematic review. Aust J Physiother 53: 19–28. 17326735 10.1016/s0004-9514(07)70058-2

[37] Schabrun SM, Hillier S (2009) Evidence for the retraining of sensation after stroke: A systematic review. Clin Rehabil 23: 27–39. 19114435 10.1177/0269215508098897

[38] Daly A, Bialocerkowski A (2009) Does evidence support physiotherapy management of adult complex regional pain syndrome type one: A systematic review. Eur J Pain 13: 339–353. 18619873 10.1016/j.ejpain.2008.05.003

[39] Viera AJ, Carrett JM (2005) Understanding inter-observer agreement: The kappa statistic. Family Medicine 37: 360–363. 15883903

[40] Fletcher J (2007) What is heterogeneity and is it important? BMJ 334: 94–96. 17218716 10.1136/bmj.39057.406644.68 PMC1767262

[41] Higgins JPT, Thompson SG, Deeks JJ, Altman DG (2003) Measuring inconsistency in meta-analyses. BMJ 327: : 557–560. Available: http://www.bmj.com/content/327/7414/557. Accessed 7 May 2014. 10.1136/bmj.327.7414.557 PMC192859 12958120

[42] Riley RD, Higgins JPT, Deeks J (2011) Interpretation of random effects meta-analyses. BMJ 342: d549 . Available: http://www.bmj.com/content/342/bmj.d549. Accessed 7 May 2014. 10.1136/bmj.d549 21310794

[43] The Cochrane Collaboration (2012) Review Manager (RevMan) [Computer program]. Version 5.2 Copenhagen: The Nordic Cochrane Centre.

[44] Herbert R (2013) Confidence Interval Calculator. Available: http://www.pedro.org.au/english/downloads/confidence-interval-calculator/. Accessed 7 May 2014.

[45] Van Tulder M, Malmivaara A, Hayden J, Koes B (2007) Statistical significance versus clinical importance: Trials on exercise therapy for chronic low back pain as example. Spine 32: 1785–1790. 17632400 10.1097/BRS.0b013e3180b9ef49

[46] Ostelo RW, de Vet HC (2005) Clinically important outcomes in low back pain. Best Pract Res Clin Rheumatol 19: 593–607. 15949778 10.1016/j.berh.2005.03.003

[47] Ostelo RW, Deyo RA, Stratford P, Waddell G, Croft P, et al (2008) Interpreting change scores for pain and functional status in low back pain: Towards international consensus regarding minimal importance change. Spine 33: 90–94. 18165753 10.1097/BRS.0b013e31815e3a10

[48] MacIntyre L (2006) The effect of Pilates on patients' chronic low back pain: A pilot study [dissertation]. Johannesburg: University of the Witwatersrand.

[49] Anderson B (2005). Randomised clinical trial comparing active versus passive approaches to the treatment of recurrent and chronic low back pain [dissertation]. Miami, Florida: University of Miami.

[50] da Fonseca JL, Magini M, de Freitas TH (2009) Laboratory gait analysis in patients with low back pain before and after a Pilates intervention. J Sport Rehabil 18: 269–282. 19561369 10.1123/jsr.18.2.269

[51] Gagnon LH (2005) Efficacy of Pilates exercises as therapeutic intervention in treating patients with low back pain [dissertation]. Knoxville: University of Tennessee.

[52] Gladwell V, Head S, Haggar M, Beneke R (2006) Does a program of Pilates improve chronic nonspecific low back pain? J Sport Rehabil 15: 338–350.

[53] Quinn J (2005) Influence of Pilates-based mat exercise on chronic lower back pain [dissertation]. Boca Raton, FL: Florida Atlantic University.

[54] Rajpal N, Arora M, Chauhan V (2008) The study on efficacy of Pilates and McKenzie exercise in postural low back pain – A rehabilitative protocol. Physiotherapy and Occupational Therapy Journal 1: 33–56.

[55] Rydeard R, Leger A, Smith D (2006) Pilates-based therapeutic exercise: Effect on subjects with nonspecific chronic low back pain and functional disability: A randomized controlled trial. J Orthop Sports Phys Ther 36: 472–484. 16881464 10.2519/jospt.2006.2144

[56] Zeada MA (2011) Ffects of Pilates on low back pain and urine catecholamine. Ovidius University Annals, Series Physiotherapy Education and Sport 12: 41–47.

[57] Rydeard RA (2001) Evaluation of a targeted exercise rehabilitation approach and its effectiveness in the treatment of pain, functional disability and muscle function in a population with longstanding unresolved low back pain [dissertation]. Kingston, Canada: Queens University.

[58] Kawanishi CY, de Oliveira MR, Coelho VS, Parreira RB, de Oliveira RF, et al (2011) Effect of Pilates exercise on trunk function and pain in patients with low back pain [Portuguese]. Revista Terapia Manual 9: 410–417.

[59] Montero-Camra J, Sierra-Silvestre E, Monteagudo-Saiz AM, Lepez-Fernandez J, Lopez-Lopez A, et al (2013) Active eccentric stretch against passive analytical hamstring stretch in subacute or chronic non-specific low back pain: A pilot trial [Spanish]. Fisioterapia 35: 206–213, doi 19.1016/j.ft.2012.10.004

[60] Dworkin RH, Turk DC, Farrar JT, Haythornthwaite JA, Jensen MP, et al (2005) Core outcome measures for chronic pain clinical trials: IMMPACT recommendations. Pain 113: 9–119. 15621359 10.1016/j.pain.2004.09.012

[61] Colle F, Rannou F, Revel M, Fermanian J, Poiraudeau S (2002) Impact of quality scales on levels of evidence inferred from a systematic review of exercise therapy and low back pain. Arch Phys Med Rehabil 83: 1745–1752. 12474181 10.1053/apmr.2002.35657

[62] Noordzij M, Tripepi Giovanni, Dekker FW, Zoccali C, et al (2010) Sample size calculations: Basic principles and common pitfalls. Nephrol Dial Transplant 25: 1388–1393. 20067907 10.1093/ndt/gfp732

[63] Stolze LR, Allison SC, Childs JD (2012) Derivation of a preliminary clinical prediction rule for identifying a subgroup of patients with low back pain likely to benefit from Pilates-based exercise. J Orthop Sports Phys Ther 42: 425–436. 22281950 10.2519/jospt.2012.3826

[64] Wells C, Kolt GS, Marshall P, Bialocerkowski A (2014) Indications, benefits, and risks of Pilates exercise for people with chronic low back pain: A Delphi survey of Pilates trained physical therapists. Phys Ther, 10.2522/ptj20130568 24700138

